# Comparative gut content analysis of invasive mosquitofish from Italy and Spain

**DOI:** 10.1002/ece3.7334

**Published:** 2021-03-15

**Authors:** Sara Pirroni, Laura de Pennafort Dezen, Francesco Santi, Rüdiger Riesch

**Affiliations:** ^1^ Department of Biological Sciences Centre for Ecology, Evolution and Behaviour Royal Holloway University of London Egham UK

**Keywords:** aquatic invertebrates, biological invasions, Europe, *Gambusia holbrooki*, Poeciliidae

## Abstract

Eastern mosquitofish (*Gambusia holbrooki*) are among the most widely introduced freshwater species globally. To gain a better understanding of feeding patterns in non‐native populations, and which local factors may influence them at the population level, we carried out gut content analysis on 163 specimens from nine invasive populations in Italy and Spain. Based on previous studies, we predicted that (a) mosquitofish are omnivores with a preference for detritus and cladocerans; (b) they display size‐ and population‐specific differences in gut morphologies and diet, with larger fish feeding more intensively over a wider range of prey items; and (c) some of the variation would be associated with differences in local environmental and climatic factors. Our results confirmed our first prediction, because mosquitofish fed on a variety of diet items, among which detritus and Cladocera dominated. However, not a single diet item was shared among all populations. Congruent with our second prediction, we further identified size‐ and population‐specific differences in the occurrence of some diet items and gut morphologies. However, observed patterns in dietary habits did not seem to be driven by the environmental and climatic variables we had quantified. The fairly variable diet likely aids invasion success and helps explain the ubiquity of invasive mosquitofish across Italy and Spain, as mosquitofish seem to be able to rely on whatever a local habitat provides. We further propose that size‐specific differences likely capture the substantial sexual size dimorphism (males are smaller than females), while population‐specific differences are likely the result of differences in local prey abundance. The lack of an influence of temperature on dietary habits suggests that mosquitofish feeding ecology may be less impacted by rising temperatures than other freshwater fish species. If true, then this suggests climate change‐induced effects may further exacerbate the competitive superiority of mosquitofish over native species in the future.

## Graphical abstract

A comparison between 9 invasive populations of eastern mosquitofish across Italy and Spain found strong size‐specific differences in feeding patterns that are probably linked to sexual dimorphism (males are smaller than females). Moreover, mosquitofish fed on a wide array of prey, yet no single diet item/category was shared among all populations. This feeding flexibility could help facilitate their invasion success.

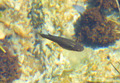

## 1 INTRODUCTION

The introduction and spread of invasive alien species (IAS) in non‐native habitats is considered among the major contributors to global change and freshwater biodiversity loss (Gkenas et al., 2012; Ricciardi & MacIsaac, 2011). For centuries, freshwater ecosystems have been subject to biological invasions at a greater extent than their terrestrial counterparts (Nunes et al., 2015; Ricciardi & MacIsaac, 2011). Currently, a total of 756 invasive alien freshwater species have been reported across Europe, with fish being the most frequently introduced aquatic taxon (Tricarico et al., 2016). The introduction of IAS to novel environments has been documented to cause a wide array of ecological effects (Ricciardi & MacIsaac, 2011). For instance, biological invasions of freshwater habitats may cause the disruption of the organization and function of native communities, which can even lead to the extinction of native biota (Carmona Catot, 2013; Mačić et al., 2018; Ricciardi & MacIsaac, 2011). Furthermore, severe economic and human health damage, as well as cascading food web effects such as changes in diet composition of native communities, have been reported to be associated with the introduction of IAS (Pimentel et al., 2000; Ricciardi & MacIsaac, 2011).

Eastern mosquitofish (*Gambusia holbrooki*, Poeciliidae) are native to the southeastern United States (Pyke, 2008) but are one of the most widely distributed freshwater species (Carmona Catot, 2013). Since the early 1900s, both eastern and western mosquitofish (*Gambusia affinis*) have been introduced worldwide as mosquito biocontrol agents for the prevention of malaria and, as a consequence, have successfully colonized over 50 countries (Carmona Catot, 2013). The negative impacts of *G. holbrooki* on native biota have led the species to be listed among the 100 most invasive species worldwide (Lowe et al., 2000). Their high reproductive potential, high dispersal capabilities, ability to live in a broad array of habitats and withstand adverse conditions such as extreme temperatures and salinities, and their broad diet have been proposed as the main factors promoting their colonization and establishment (Dirnberger & Love, 2016; Pyke, 2005).

Dietary studies based on stable isotope and stomach content analyses show that eastern mosquitofish feed on a variety of items, ranging from detritus to organisms such as insects, aquatic invertebrates, algae, and fish and amphibian eggs and larval stages (Macdonald & Tonkin, 2008; Pyke, 2005; Singh & Gupta, 2010). They are therefore considered adaptable omnivores or even generalist predators, capable of changing the composition of their diet depending on food availability (Macdonald & Tonkin, 2008). This variability of their diet also has an impact on gut fullness and gut morphologies (i.e., relative gut length) as well as niche breadth, which differ in relation to their diet and fish size. Specifically, larger specimens often exhibit broader diets and lower levels of gut fullness than smaller fish, and shorter guts are associated with a more carnivorous diet (i.e., more invertebrates), whereas longer guts correspond to a more herbivorous diet (i.e., more detritus and plant material) (Blanco et al., 2004; Rehage et al., 2005; Singh & Gupta, 2010). Moreover, shifts in dietary composition and niche breadth have also been documented to occur depending on season, geographic location and time of the day (Gkenas et al., 2012; Macdonald & Tonkin, 2008; Pyke, 2005; Specziár, 2004). Furthermore, some studies have shown that water temperature as well as other environmental characteristics such as nutrient concentration and pH can also influence their feeding rates (i.e., the number of prey items caught) and diet diversity directly or through indirect effects on aquatic biodiversity (Blanco et al., 2004; Cabral et al., 1998; Oliver, 1991).

Although extensive research has been carried out on the feeding ecology of *Gambusia* in general, with some dietary studies focused on the role of mosquitofish in controlling zooplankton assemblages (Blanco et al., 2004; Peck & Walton, 2008), so far, studies on dietary patterns of eastern mosquitofish in their invasive range focused only on very specific locations, on small geographic scales (Blanco et al., 2004; Cabral et al., 1998; Erguden, 2013; Singh & Gupta, 2010). Furthermore, to date there is still scarcity of data on how habitat characteristics might impact on feeding ecology and, to our knowledge, only one study (Cabral et al., 1998) investigated the potential association between habitat features (e.g., vegetation coverage) and the amount of prey eaten. Yet, knowledge of the feeding habits of this species (including potential predator–prey interactions under different environmental conditions) and the role of associated habitat characteristics is crucial for understanding food web dynamics and resource partitioning and to identify appropriate management and control strategies for this highly invasive freshwater species.

To get a more complete picture of variability in dietary habits across the European invasive range, we sampled *G. holbrooki* from nine distinct populations across a large geographic area in Italy and Spain. Specifically, we made an effort to sample from a diverse range of habitats (i.e., drainage ditches, lakes, rivers) and covering as much geographic distance as possible to attempt to better capture the full extent of variation in feeding habits across the invasive range. We aimed to assess: (a) the general variability of their diet; (b) the size‐specific and population‐specific differences in diets and gut morphologies (e.g., to what extent do large and small specimens differ in the type of food they consume); and (c) whether differences in environmental and climatic parameters between populations explain any differences observed in their diet or gut morphologies (i.e., diet diversity, frequency of occurrence of diet items and their relative importance, but also length and fullness of the guts of the specimens). Based on previous research (Blanco et al., 2004; Cabral et al., 1998; Erguden, 2013; Sánchez‐Hernández et al., 2012; Singh & Gupta, 2010), we predicted that mosquitofish (a) would be omnivores with a heavy reliance on detritus and cladocerans; (b) would display size‐ and population‐specific differences in gut morphologies and diet, with larger specimens feeding over a wider range of prey items; and (c) that some of the population differences would be associated with differences in local environmental and climatic factors. To our knowledge, this is the first study that aims to make a direct comparison of the dietary patterns of natural populations of this species across such a large geographic area across two countries in its invasive European range.

## 2 METHODS

### 2.1 Field sampling

**FIGURE 1:**
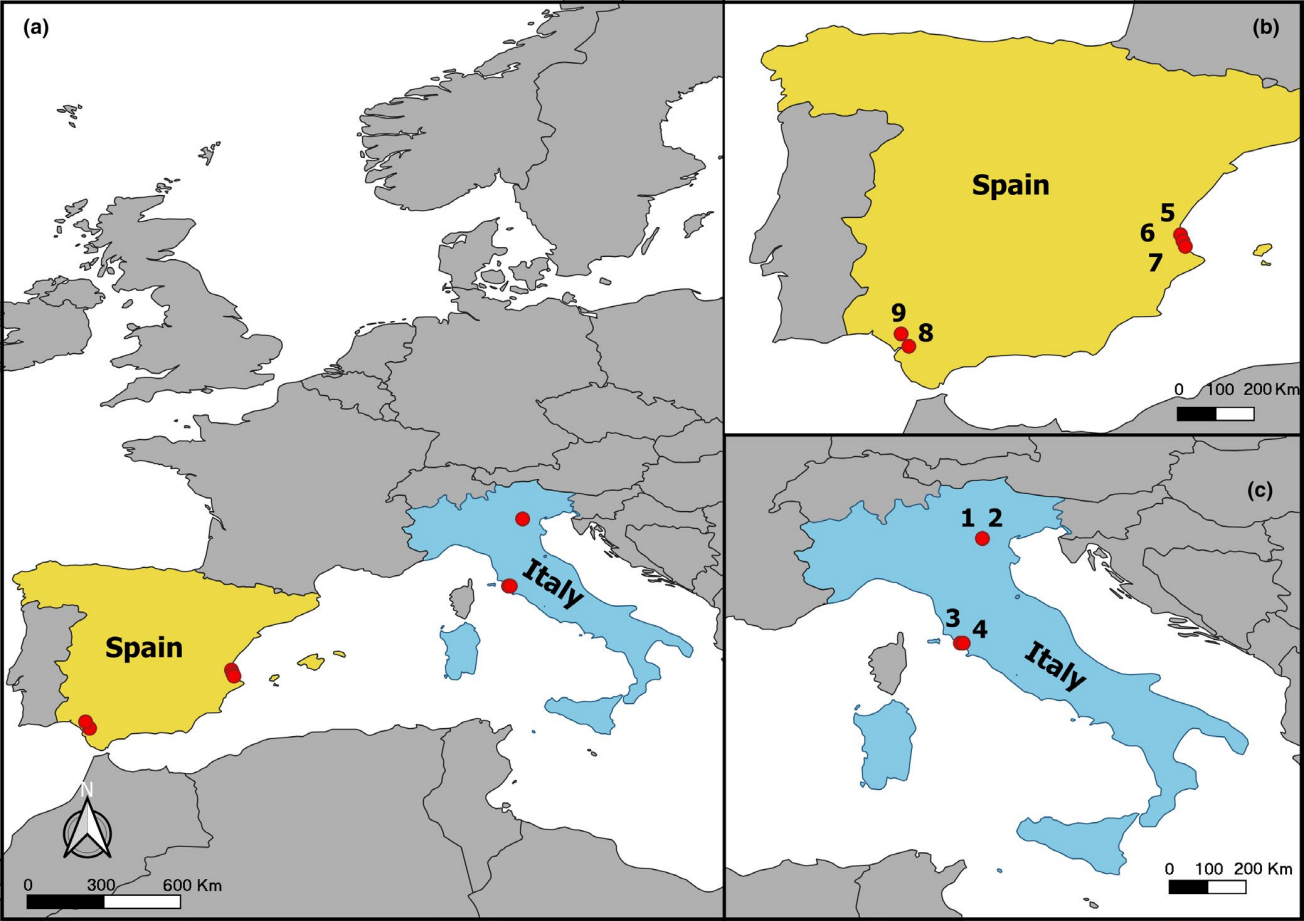
Map of the sampling sites of *Gambusia holbrooki* in Europe (a), with the subpanels showing only sample sites in Spain (b) and Italy (c). The map was generated using QGIS 3.2

Fieldwork was performed during a 15‐day period between 27 July and 10 August 2017. A total of 163 live specimens of *G. holbrooki* were collected with dip nets (2 mm mesh size) from nine sites in Italy and Spain, spanning ca. 8° latitude and 18° longitude, in order to assess geographic variation in feeding habits and investigate the influence of associated environmental characteristics (Figure 1, Appendix A: Table A1). Sampling sites were aquatic habitats with slow current or stagnant water and dense riparian vegetation. Immediately upon capture, fish were sacrificed with clove oil and then preserved in 96% ethanol for subsequent analyses. Conductivity (mS/cm), dissolved oxygen (mg/L), pH, and water temperature (°C) were measured in situ at each site using a Hach Rugged DO/pH/Conductivity Field Kit (Hach, Loveland, Colorado, USA). Four climatic variables were additionally downloaded from the European Climate Assessment and Dataset (ECA&D) ver. 20.0e (Cornes et al., 2018) database at 0.1 degrees resolution: daily mean temperature, daily maximum temperature, daily minimum temperature, and daily precipitation sum (Appendix A: Table A1).

### 2.2 Gut content analysis

In the laboratory, fish were sexed based on the presence/absence of the male copulatory organ, the gonopodium (i.e., the modified anal fin; Pyke, 2005), and were measured for standard length (SL) using a digital caliper (to the nearest 0.01 mm). The body cavity was opened, and the entire intestinal tract of all mosquitofish was excised. Total gut length was then also measured with the caliper (again to the nearest 0.01 mm), and afterward, gut contents were removed and examined using a dissecting microscope. Gut fullness and relative gut length (relative gut length = gut length/SL) were calculated for each specimen. When present, prey items were counted and identified to the lowest possible taxon. This resulted in the following prey categories: Bivalvia, Branchiopoda, Araneae, Cladocera, Coleoptera, Diptera, Entognatha, Hemiptera, Hymenoptera, and Ostracoda. Prey items that were too digested to be physically separated for accurate identification were classified as unidentifiable.

The dietary importance of each prey category was estimated by quantifying their frequency of occurrence and the index of relative importance following Singh and Gupta (2010) and Hyslop (1980), respectively. Prey diversity in the diet was quantified using the Shannon–Wiener index, which accounts for both abundance and evenness of the prey items. The degree of individual diet specialization (relative niche width) was estimated as the proportion of the food categories in the diet of each specimen relative to the total number of food categories. Relative niche width values vary from 0 (specimen consumed items belonging to a single category) to 1 (specimen exploited all prey categories). While we could not include detritus in our calculation of the index of relative importance or the Shannon–Wiener index (i.e., we could easily determine relative gut volume taken up by detritus but not really “count” detritus in the same way as was possible for prey items), we included detritus in our quantification of frequency of occurrence (presence/absence) and relative niche width. For equations relating to these indices, please refer to Appendix A: Table A2.

To evaluate dietary overlap between populations, we further calculated the index of overlap (again excluding detritus due to a lack of count data), as proposed by Schoener (1970). This measure of overlap ranges from 0 (absence of overlap) to 1 (complete overlap in resource use) and according to Wallace and Ramsey (1983), values higher than 0.6 are considered as biologically significant overlap. To further validate the significance of these overlaps, all values were compared to the overlap values obtained using Pianka's diet overlap index (Pianka, 1980). Due to logistical constraints in the field, it was not possible to collect data on resource availability at each site, and consequently, overlap values were calculated based on the assumption that the different dietary resources were equally accessible to all populations. Again, please refer to Appendix A: Table A2 for details.

### 2.3 Statistical analysis

All statistical analyses were performed using the software R x64 3.5.1 (R Development Core Team, 2019) except for principal components analysis, which was performed using IBM SPSS Statistics 25.0 (IBM Inc.).

#### 2.3.1 Feeding patterns as a function of size and population

As members of the family Poeciliidae, eastern mosquitofish display a pronounced sexual size dimorphism, because males are significantly smaller than females (Bisazza, 1993). This was supported in our data (female SL, mean ± *SD*: 21.5 ± 3.5 mm (range: 10.1–37.5 mm); male SL: 17 ± 2.5 mm (11.4–25.7 mm)). Therefore, sex and SL in our dataset were strongly correlated (*η* ^2^ = 0.962), resulting in a violation of model assumptions (i.e., no multicollinearity) if we wanted to include both in the same model. Moreover, the inclusion of both also led to a significant loss in statistical power which we uncovered during preliminary data screening. We therefore decided to only consider SL for subsequent analyses.

To investigate whether the occurrence of a food item in the diet was influenced by the size of the fish or the sampling site, we applied food category‐specific generalized linear models (GLMs) with a binomial error distribution and a logit link function. In all models, we included the presence/absence of an individual food category in the diet of individual fish (i.e., individuals feeding on the 10 prey categories mentioned above as well as those feeding on detritus, resulting in 11 separate models) as the response variable, and SL, population, and the interaction “SL‐by‐population” as factors. Interaction terms were removed from the model when they were associated with a *p* > .2, and the model was then refitted with the remaining parameters. All models were fitted using the MASS R package (v7.3–51.5; Venables & Ripley, 2002), and diagnostic plots of residuals were checked for appropriate model fitting prior to consideration of estimated model parameters.

Furthermore, to assess the effects of SL and sampling site on fish relative niche width, gut fullness and relative gut length, we used GLMs with a gamma error distribution and log link function. The models were initially fitted with relative niche width, gut fullness, and relative gut length serving as response variables, and SL, population, and interaction term “SL‐by‐population” as factors. If the interaction term was *p* > .2, the interaction term was removed from the models and the models refitted with the remaining parameters. When we found significant effects, we ran *post hoc* univariate models separately for each response variable to identify whether significant multivariate effects were due to significant effects on all or only some response variables. Each model was applied after having checked for model validation and overdispersion. A Tukey's HSD multiple comparison test was also performed to determine whether there was a difference between the mean values of relative niche width, gut fullness, and relative gut length of all population pairs, and by using the multcomp R package (v1.4‐13; Hothorn et al., 2008).

#### 2.3.2 Effects of environmental, geographic, and climatic variables on diet composition

A univariate approach was used to investigate whether population differences in diet [i.e., differences in diet diversity (Shannon–Wiener index) and dietary importance of food items (frequency of occurrence and index of relative importance)] could be partially explained by the effects of the environmental, geographic, and climatic parameters associated with each sampling site (Appendix A: Table A1). First, we performed a principal component analysis (PCA) on the matrix of environmental, geographic, and climatic data, and retained the first four axes (PCs), which explained over 90% of the total variance (Appendix A: Table A3). Linear regression models were then performed separately for different input variables: Shannon–Wiener index, index of relative importance, and frequency of occurrence as dependent variables and using the four PCs as covariates.

Because previous research had revealed that the total number of prey ingested by eastern mosquitofish was often correlated with environmental and habitat features such as water temperature and area covered by aquatic vegetation (Alison & Cech, 1990; Cabral et al., 1998), we examined our data for similar patterns by calculating Pearson's correlation coefficients between the total amount of prey consumed by each population and the four PCs.

We used the same approach to test whether these variables influenced the proportion of specimens with empty guts in each population.

Finally, we examined whether interpopulation dietary overlap between pairs of populations was associated with the geographic distance between them. This was accomplished by developing a pairwise geographic distance matrix for all sampling sites based on their GPS coordinates and comparing this against the pairwise matrix with Shoener overlap index values (see above) using a Mantel test with 10,000 permutations fitted with the ade4 R package (v1.7–15; Dray & Dufour, 2007). We did not use Pianka's overlap values for this analysis, given that a very strong correlation (*p* < .001, *r* = 0.85) was found between both overlap indices.

## 3 RESULTS

### 3.1 Gut composition and dietary overlaps

**FIGURE 2:**
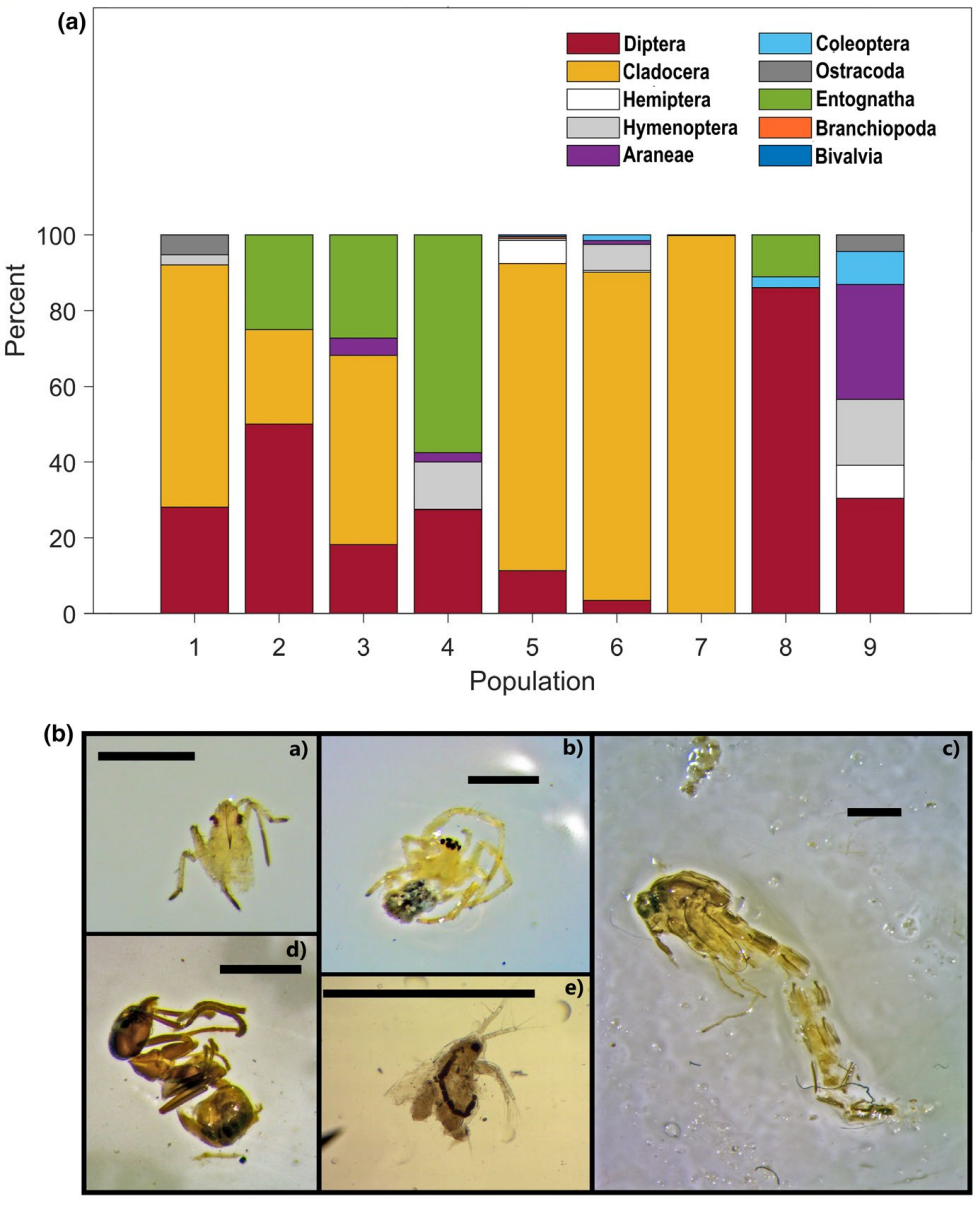
(A) Dietary compositions of nine populations of eastern mosquitofish; (B) typical prey items found in fish guts: (a) Hemiptera; (b) Araneae; (c) Diptera; (d) Hymenoptera; and (e) Cladocera. Pictures were stacked using Helicon Focus 7.5.8 (Helicon Soft Ltd. 2000) Scale bars represent 1 mm

A summary of the gut contents of 86 female and 77 male *G. holbrooki* across all nine populations is provided in Table 1 and Figure 2. Mean relative gut length was greater for females compared to males (mean ± *SD*, females: 0.6 ± 0.2 mm, males: 0.5 ± 0.1 mm). Of the total number of guts examined (*n* = 163), 17.8% were completely empty and 38% contained only detritus (e.g., sediment material and plant debris) or unidentifiable food items. Sampled populations consumed similar prey items, although in different proportions. The most common overall food category was detritus, which we found in individuals from all but one population (P1). Cladocera were overall the most abundant prey category, comprising 86.9% of the total diet of all mosquitofish analyzed, and consumed by fish in all but three populations (Table 1, Figure 2). Dipterans, such as hatching mosquito larvae belonging to the family Culicidae, were the second most common prey item (6.9%) and were consumed by fish in all but one population (P7; Figure 2). The contribution of the other prey categories to the overall diet was negligible, although on the population level they often occurred at a high frequency. For instance, Entognatha and terrestrial Araneae constituted 1.9% and 0.7% (respectively) of the overall diet of sampled specimens, but Entognatha constituted 57.5% of the diet of population 4, while Araneae composed 30.4% of the diet of population 9.

**TABLE 1:** Composition of the gut contents of Gambusia *holbrooki* (from Italian and Spanish populations) by frequency of occurrence (FO) and index of relative importance (IRI), with Shannon‐Wiener diversity index (*H*) and mean relative niche width (RNW) provided as well

Population	Detritus		Prey categories																				Shannon–Wiener index (*H*)	Mean relative niche width (RNW)	Relative gut length (RGL)		Gut fullness (GF)	
			BI (aquatic)		BRA (aquatic)		ARA (terrestrial)		CLA (aquatic)		COL (terrestrial)‐‐		DIP (terrestrial and semi‐aquatic)		ENT (semi‐aquatic)		HEM (terrestrial and aquatic)		HYM (terrestrial)		OST (aquatic)				SEX		SEX	
	FO	IRI	FO	IRI	FO	IRI	FO	IRI	FO	IRI	FO	IRI	FO	IRI	FO	IRI	FO	IRI	FO	IRI	FO	IRI			F	M	F	M
Italy																												
P1	–	–	–	–	–	–	–	–	43.7	8,777.8	–	–	50	6,898.6	–	–	–	–	18.7	923.1	25	3,756.6	0.356	0.138	0.6 ± 0.1	0.4 ± 0.1	0.3 ± 0.3	0.3 ± 0.3
P2	47.4	–	–	–	–	–	–	–	5.3	184.2	–	–	5.3	289.5	5.3	289.6	–	–	–	–	–	–	0.346	0.049	0.4 ± 0.1	0.4 ± 0.1	0.1 ± 0.2	0.0 ± 0.0
P3	52.6	–	–	–	–	–	5.3	287.1	57.9	4,921.1	–	–	21.1	3,645.9	31.6	3,703.3	–	–	–	–	–	–	0.309	0.075	0.5 ± 0.1	0.4 ± 0.1	0.2 ± 0.2	0.2 ± 0.3
P4	47.8	–	–	–	–	–	4.4	206.5	–	–	–	–	30.4	3,423.9	43.5	12,934.8	–	–	21.4	4,293.5	–	–	0.355	0.067	0.5 ± 0.2	0.5 ± 0.1	0.4 ± 0.2	0.3 ± 0.3
Spain																												
P5	61.1	–	5.5	85.9	5.5	91.5	–	–	55.5	20,917.8	–	–	6.2	142.4	–	–	22.2	2,504.5	5.5	91.5	–	–	0.356	0.197	0.8 ± 0.1	0.6 ± 0.2	0.7 ± 0.2	0.4 ± 0.2
P6	25	–	–	–	–	–	10	434.8	50	18,374.9	15	1,384	30	6,577.5	–	–	5	177.5	35	6,422.4	–	–	0.346	0.053	0.5 ± 0.1	0.4 ± 0.1	0.5 ± 0.2	0.4 ± 0.4
P7	25	–	–	–	–	–	5	1,135.4	90	90,564.1	–	–	–	–	–	–	–	–	–	–	5	1,135.3	0.309	0.058	0.5 ± 0.1	0.4 ± 0.1	0.6 ± 0.3	0.5 ± 0.2
P8	31.3	–	–	–	–	–	–	–	–	–	6.2	142.4	56.1	4,066.3	25	1797.8	–	–	–	–	–	–	0.355	0.052	0.7 ± 0.1	0.5 ± 0.1	0.2 ± 0.3	0.2 ± 0.2
P9	58.3	–	–	–	–	–	50	8,766.7	–	–	16.7	838.2	41.7	9,843.1	–	–	16.7	223.7	33.3	6,606.3	8.3	223.7	0.356	0.157	0.6 ± 0.2	0.4 ± 0.2	0.6 ± 0.2	0.2 ± 0.2

Note

Prey items belonged to the following prey categories: Bivalvia (BI), Branchiopoda (BRA), Araneae (ARA), Cladocera (CLA), Coleoptera (COL), Diptera (DIP), Entognatha (ENT), Hemiptera (HEM), Hymenoptera (HYM), and Ostracoda (OST).

For 63% of the population pairs, diet overlap values were moderate to high (>0.6), suggesting the exploitation of similar food resources among different populations (Table 2). Nonetheless, mean Shannon–Wiener index and relative niche width values of sampled populations varied from 0.309 to 0.356 and 0.049 to 0.197, respectively, indicating that most populations had relatively limited niches (Table 1). Furthermore, we found no evidence that dietary overlap between population pairs was a function of geographic distance (Mantel test: *r* = −0.11, *p* = .78).

**TABLE 2:** Pianka (lower triangular matrix; white background) and Shoener (upper triangular matrix; gray background) index values of dietary overlap between our nine sampling sites. Indices > 0.6 in bold

	P1	P2	P3	P4	P5	P6	P7	P8	P9
P1	–	0.53	**0.68**	0.30	**0.75**	**0.70**	**0.64**	0.28	0.35
P2	**0.70**	–	**0.68**	0.52	0.36	0.28	0.25	**0.61**	0.30
P3	**0.88**	**0.77**	–	0.48	**0.61**	0.54	0.5	0.29	0.23
P4	0.18	**0.71**	0.53	–	0.12	0.11	0	0.39	0.42
P5	**0.96**	0.52	**0.86**	0.06	–	**0.86**	**0.81**	0.11	0.18
P6	**0.93**	0.28	**0.84**	0.03	**0.99**	–	0.13	0.05	0.13
P7	**0.91**	0.41	**0.83**	0	**0.99**	0.06	–	0	0
P8	0.40	**0.86**	0.36	0.53	0.14	0.04	0	–	0.33
P9	0.27	0.51	0.24	0.36	0.10	0	0	**0.63**	–

### 3.2 Feeding patterns in relation to SL and population

**FIGURE 3:**
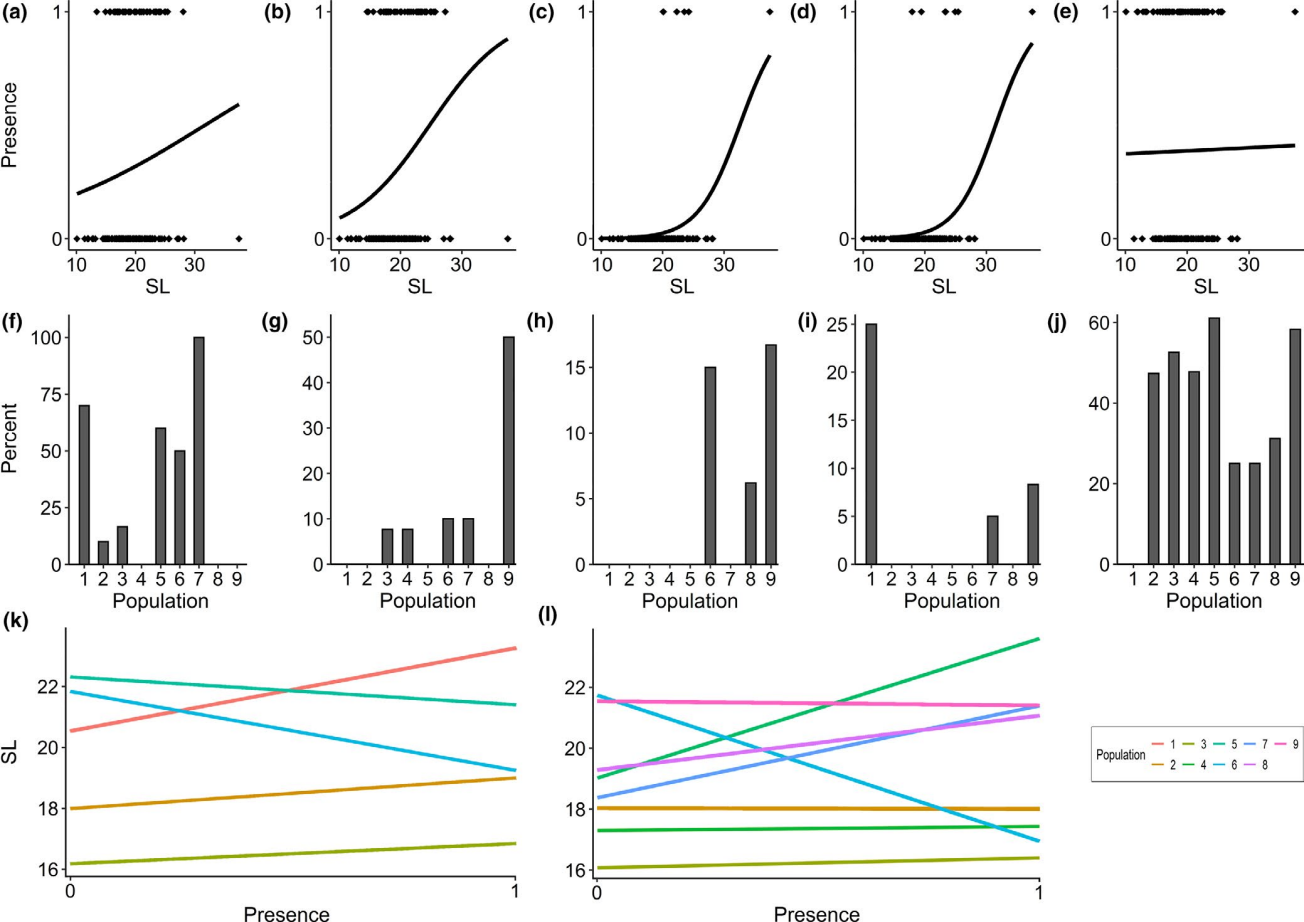
Presence–absence plots for (a) Cladocera, (b) Diptera, (c) Ostracoda, (d) Hemiptera, and (e) detritus; bar graphs displaying interpopulation differences for (f) Cladocera, (g) Araneae, (h) Coleoptera, (i) Ostracoda, and (j) detritus; plots displaying the significant SL‐by‐population interaction for (k) Cladocera and for (l) detritus

With respect to the presence or absence of certain diet items, we found that SL, population, and their interaction significantly affected the occurrence of Cladocera and detritus, SL and population also had a significant effect on the occurrence of Ostracoda, SL had a significant influence on the occurrence of Diptera and Hemiptera, and population had a significant influence on the occurrence of Araneae and Coleoptera (Table 3). Specifically, larger fish were more likely than smaller specimens to have eaten these food categories (size effects; Figure 3a–e). Also, the occurrence of these food categories differed between populations (population effects; Figure 3f–j). For example, Cladocera were common in the diet of populations 1, 5, 6, and 7, but rare in populations 2 and 3, and absent from the diet in populations 4, 8 and 9 (Figure 3f). For detritus, a greater proportion of specimens from populations 2, 3, 4, 5 and 9 had ingested detritus compared to fish belonging to populations 6, 7, and 8; and detritus was absent from the guts of population 1 (Figure 3j). However, for Cladocera and detritus, these effects were not independent from each other (SL‐by‐population interaction effect). The presence of Cladocera in the diet increased with SL in Italian populations (P1‐P3), but decreased with SL in Spanish populations (P5 and P6; Figure 3k). Conversely, the presence of detritus in the diet increased with SL for all but three populations (P2, P6, and P9; Figure 3l).

**TABLE 3:** Parameter estimates of GLMs with binomial family investigating the influence of the population and the SL of *G. holbrooki* on the presence of prey items (including detritus) in their guts

	Estimate	*SE*	*z*	*p*
Cladocera				
Intercept	−9.400	2.448	−3.840	**<.001**
SL	0.403	0.120	3.370	**<.001**
Population	0.744	0.208	3.572	**<.001**
SL × Population	−0.034	0.010	−3.324	**<.001**
Diptera				
Intercept	−2.976	0.647	−4.600	**<.001**
SL	0.083	0.032	2.631	**<.001**
Population	0.019	0.024	0.776	.437
[SL × Population]	[−0.007]	[0.006]	[−1.182]	[.237]
Entognatha				
Intercept	1.463	1.383	1.057	.290
SL	−0.166	0.078	−2.129	.332
Population	−0.054	0.045	−1.176	.239
[SL × Population]	[0.012]	[0.013]	[0.982]	[.326]
Hymenoptera				
Intercept	−5.105	1.329	−3.839	**<.001**
SL	0.148	0.064	2.308	.021
Population	0.015	0.042	0.363	.716
[SL × Population]	[−0.010]	[0.011]	[−0.928]	[.353]
Araneae				
Intercept	−5.367	1.976	−2.715	**<.001**
SL	−0.001	0.083	−0.014	.988
Population	0.224	0.090	2.472	**.013**
[SL × Population]	[0.023]	[0.197 ]	[1.156]	[.247]
Coleoptera				
Intercept	−8.201	2.753	−2.979	**<.01**
SL	0.062	0.093	0.671	.502
Population	0.289	0.147	1.969	**.049**
[SL × Population]	[−0.002]	[0.032]	[−0.064]	[.949]
Ostracoda				
Intercept	−10.956	2.957	−3.706	**<.001**
SL	0.423	0.136	3.105	**<.001**
Population	−0.214	0.100	−2.157	**.031**
[SL × Population]	[0.002]	[0.018]	[0.126]	[.899]
Branchiopoda				
Intercept	−10.278	3.993	−2.574	**.010**
SL	0.231	0.181	1.281	.200
Population	0.017	0.202	0.083	.934
[SL × Population]	[−0.111	[0.120]	[−0.921]	[.357]
Bivalvia				
Intercept	−9.062	4.122	−2.199	**.028**
SL	0.164	0.186	0.882	.377
Population	0.051	0.198	0.255	.798
[SL × Population]	[−0.038]	[0.072]	[−0.531]	[.596]
Hemiptera				
Intercept	−10.522	2.822	−3.729	**<.001**
SL	0.273	0.114	2.387	**.017**
Population	0.138	0.100	1.383	.167
[SL × Population]	[−0.015]	[0.031]	[−0.474]	[.636]
Detritus				
Intercept	2.884	1.690	1.706	.088
SL	−0.185	0.091	−2.032	**.042**
Population	−0.372	0.166	−2.244	**.025**
SL × Population	0.020	0.008	2.367	**.018**

Note

When a highly nonsignificant effect of the interaction “SL‐by‐population” was found (*p* > .2), the interaction term was removed from the model and the model refitted with the remaining parameters; this is indicated with the interaction term provided in brackets. Significant *p*‐values in bold.

**FIGURE 4:**
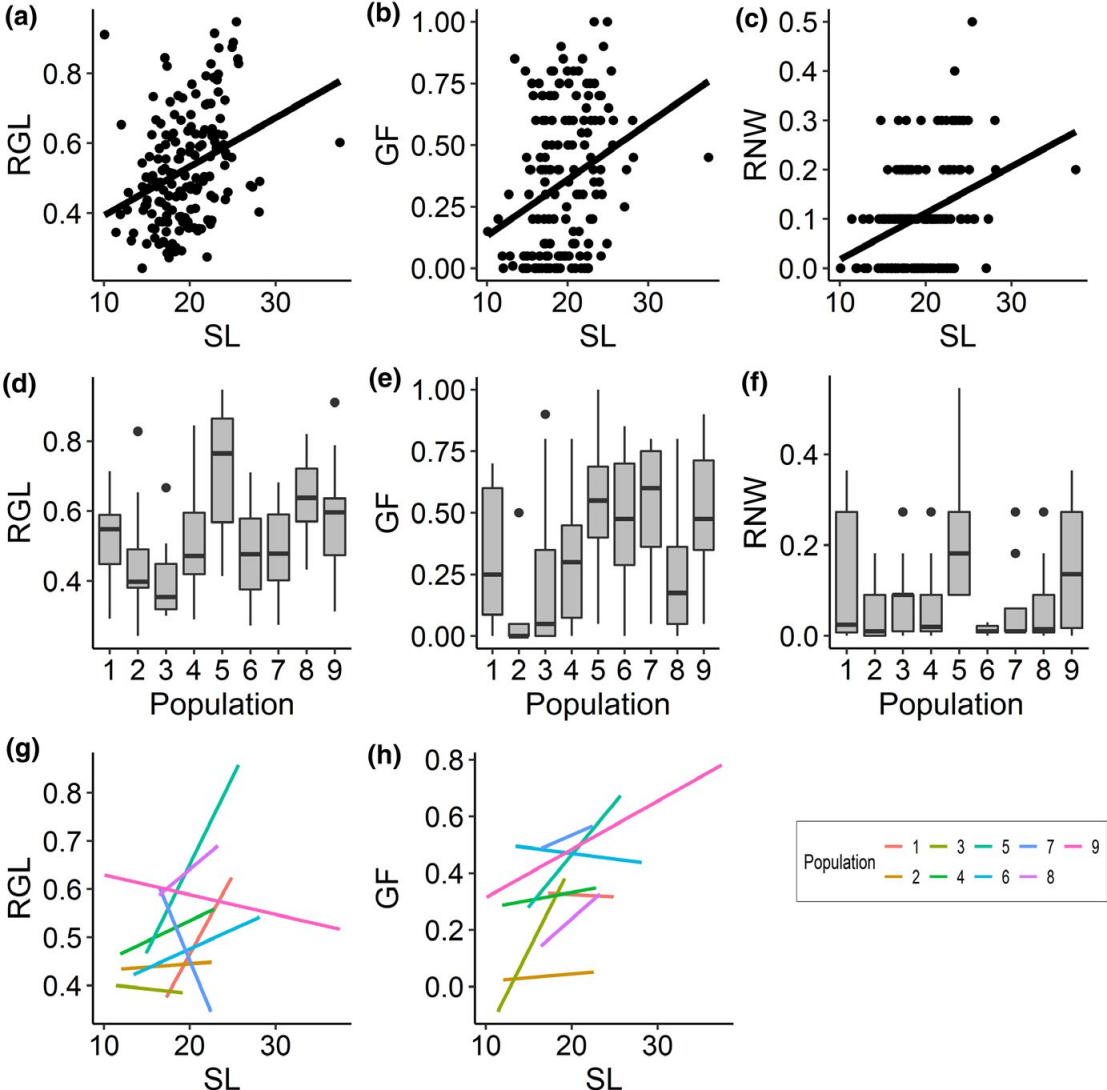
Scatter plots displaying the relationship between (a) relative gut length (RGL), (b) gut fullness (GF), (c) relative niche width (RNW), and fish SL; box plots with outliers displaying the significant population‐specific differences in (d) relative gut length and (e) gut fullness, and the nonsignificant trend for a population effect for (f) relative niche width; (g–h) plots depicting the significant SL‐by‐population interaction effect on these parameters

Finally, in our multivariate GLM with gamma family we discovered significant effects of SL, population, and their interaction on combined dependent variables (relative gut length, gut fullness, and relative niche width). Post hoc univariate GLMs revealed that SL, population, and their interaction significantly affected fish relative gut length and gut fullness, while only SL significantly influenced fish relative niche width and there was a trend for a population effect for this latter variable (Table 4). Specifically, smaller fish had shorter relative gut lengths, narrower niche widths, and lower values of gut fullness than larger specimens (Figure 4a–c), and these traits also differed between populations (Figure 4d–f). For instance, specimens belonging to population 5 exhibited significantly longer guts than fish belonging to all but two populations (Figure 4d; Appendix A: Table A4). On the other hand, fish from population 2 had significantly lower values of gut fullness compared to the other populations, whereas fish belonging to population 6 had smaller niches than specimens belonging to all populations, albeit this difference was not significant (Figure 4e;f; Appendix A: Tables A5 and A6). Furthermore, the significant interaction effects of SL‐by‐population for fish relative gut length and gut fullness indicated that both traits scaled differently with body size across populations. Specifically, relative gut length increased with SL for six out of nine populations, whereas this pattern was reversed for populations 3, 7, and 9 (Figure 4g). Moreover, relative gut fullness decreased with fish size in specimens from populations 1 and 6, while all other populations exhibited a positive association between the two variables (Figure 4h).

**TABLE 4:** Parameter estimates of GLMs with gamma family investigating the influence of the population, the SL of *G. holbrooki* and the interaction “*SL*‐by‐population” on their gut fullness (GF), relative gut length (RGL), and relative niche width (RNW)

	Estimate	*SE*	*z*	*p*
Multivariate model				
Intercept	−2.106	0.384	−5.482	**<.001**
SL	−0.89	0.020	4.597	**<.001**
Population	0.242	0.068	3.548	**<.001**
SL × Population	−0.009	0.003	−2.681	**<.001**
RGL model				
Intercept	−1.905	0.254	−7.500	**<.001**
SL	0.057	0.013	4.429	**<.001**
Population	0.155	0.045	3.451	**<.001**
SL × Population	−0.006	0.002	−2.888	**<.001**
GF model				
Intercept	−3.871	0.723	−5.353	**<.001**
SL	0.122	0.037	3.325	**<.01**
Population	0.364	0.128	2.840	**<.01**
SL × Population	−0.013	0.010	−2.066	**.040**
RNW model				
Intercept	−2.736	0.301	−9.097	**<.001**
SL	0.045	0.015	2.984	**<.01**
Population	−0.043	0.023	−1.864	.064
[SL × Population]	[0.004]	[0.006]	[0.640]	[.523]

Note

Significant values are highlighted in bold. When a highly nonsignificant effect of the interaction “SL‐by‐population” was found (*p* > .2), the interaction term was removed from the model and the model refitted with the remaining parameters; this is indicated with the interaction term provided in brackets.

### 3.3 Influence of environmental, geographic, and climatic variables on population differences in diet

There were no effects of environmental, geographic, or climatic variables on Shannon–Wiener index, index of relative importance, and frequency of occurrence between populations (*p* ≥ .2 in all cases; Appendix A: Table A7). Similarly, there was no impact of these variables on the total number of prey ingested by each population or the proportion of empty guts (*p* ≥ .098 in all cases; Tables A8 and A9).

## 4 DISCUSSION

We investigated the feeding ecology of 163 specimens of *G. holbrooki* sampled from invasive Italian and Spanish populations. Our analysis of gut contents confirmed that eastern mosquitofish are generalist feeders, with slight preferences for some food items, such as detritus and cladocerans, and we uncovered some size‐ and population‐specific differences in their diet. Furthermore, we found moderate‐to‐high dietary overlaps between sampled populations, indicating the exploitation of similar food resources. Nonetheless, although we found population‐specific feeding patterns, there was no evidence that these were associated with our environmental, geographic, or climatic parameters.

### 4.1 Food habits and dietary overlaps

In accordance with previous studies on the dietary patterns of eastern mosquitofish (Blanco et al., 2004; Cabral et al., 1998; Gkenas et al., 2012; Singh & Gupta, 2010), our results confirmed that *G. holbrooki* are omnivores that feed on a wide variety of food items. Moreover, our study suggests that mosquitofish in both the native and invasive range essentially feed on the same set of diet items, albeit in different proportions. For instance, a study of Dirnberger and Love (2016) on native *G. holbrooki* from a population in Georgia reported a preference for dipteran larvae and pupae over other taxa, while Cladocera were also commonly preyed upon. In support of our prediction 1, detritus and Cladocera were widely consumed also in our data. For example, detritus being consumed by 61% of the specimens of one population (population 5) and Cladocera accounting for more than 80% of the total diet of all fish analyzed. Our dissection results are likely to derive from a direct preference for these food categories, as previous experimental studies of García‐Berthou (1999), Blanco et al. (2004) and Singh and Gupta (2010) have reported a preference by *Gambusia* for both. The preference for Cladocera has been ascribed to *Gambusia* being morphologically adapted to forage at and near the water surface (Macdonald & Tonkin, 2008; Pyke, 2005; Singh & Gupta, 2010). However, Cabral et al. (1998) reported that free‐living Cyclopoid copepods were the dominant prey group of eastern mosquitofish caught in rice fields in Portugal. A possible explanation for these different findings could be the differential availability of prey species in different habitats. Congruent with this explanation, we found no cladocerans in the diet of three of our nine populations.

We also observed that, although mosquito larvae and pupae, and other dipterans were the second most important group, and were consumed by all but one population, they constituted only a relatively small proportion of mosquitofish’ overall diet. This is further evidence (see also Singh & Gupta, 2010) that, despite the common name mosquitofish, mosquitoes are not the main prey item for this invasive species. In line with this result, Pyke (2005) reported that their efficiency for mosquito control remains questionable.

Surface insects, terrestrial arthropods, aquatic hemiptera, such as water boatmen and backswimmers, and Ostracoda, such as seed shrimps, were additional food items for mosquitofish from Italy and Spain. However, they formed a negligible proportion of the overall diet of all fish. These taxonomic groups have also been reported to be part of the diet of *G. holbrooki* in previous studies (Blanco et al., 2004; Gkenas et al., 2012; Singh & Gupta, 2010; Specziár, 2004). The low numbers of Ostracoda in the diet of the sampled specimens may be attributable to the fact that most of the crustaceans belonging to this category are benthic and therefore less accessible to mosquitofish compared to other groups of crustaceans that are mostly planktonic (Crivelli & Boy, 1987). Nonetheless, because detrital material was found in many of the guts examined, it is also plausible that many ostracods were too digested to be accurately quantified and were thus accidentally counted as “detritus.” However, the presence of detritus in the diet of our specimens is congruent with several previous studies (e.g., Blanco et al., 2004; García‐Berthou, 1999; Specziár, 2004; Yoğurtçuoğlu & Ekmekçi, 2017), in some of which more than 50% of the gut contents examined were found to be consisting of detritus. This reliance on detritus might be due to one of three not mutually exclusive reasons. First, it is possible that detritus is simply accidentally ingested, while *G. holbrooki* are making a predation attempt on invertebrates close to, or on, the sediment (detritus is one of the main food sources of many invertebrates that mosquitofish prey on; Blanco et al., 2004). Second, detritus may also derive from digested material that were too decomposed to be discriminated from detritus. Third, it is possible that mosquitofish who had been unsuccessful in their pursuit of live prey for a while turn to active detritus consumption as an emergency means of acquiring energy (Blanco et al., 2004). Irrespective of the factors driving the presence of detritus in mosquitofish diet, our results indicate that eastern mosquitofish in a large variety of different habitats and geographic locations are highly omnivorous with a diet that is often dominated by detritus.

Even though most of the diet items occurred in the diet of each population, their relative proportion differed greatly. While these findings suggest the exploitations of similar food resources among populations, the low mean values of the Shannon–Wiener index and relative niche width indicate that most populations had relatively small dietary niches. As previously mentioned, one explanation for the high dietary overlap might be because we investigated this based on the assumptions that the food items (particularly prey consumed) were equally accessible to all populations, and without having estimates of the real availability of these prey resources in the habitats. Furthermore, our findings might be influenced by the presence of many partially digested and unidentifiable food items, making it difficult to get the whole picture of the diet overlaps between populations.

Interestingly, two other studies (Gkenas et al., 2012; Yoğurtçuoğlu & Ekmekçi, 2017), who studied a *G. holbrooki* population in Lake Pamvotis in Greece, and the wetlands surrounding Lake Acigöl in Turkey, reported a broader trophic niche for their populations, with Shannon–Wiener indices ranging between 0.46 and 0.68 (Gkenas et al., 2012) and 2.01 and 2.66 (Yoğurtçuoğlu & Ekmekçi, 2017) across sites and seasons (while it varied between 0.309 and 0.356 in our study). These differences might be attributable to the fact that both studies generally worked with larger sample sizes for their individual collections, making it more likely that rare diet items turned up in their, relative to our dataset; this would result in higher diversity indices. However, it is also possible that both habitats in Greece and Turkey simply provide a larger variety of potential prey for *G. holbrooki* than the populations we sampled in Italy and Spain. This latter explanation could be due to a variety of reasons, ranging from differential pressures of predation and competition to differential availability of resources between their and our sampled populations.

### 4.2 Influence of SL and population on feeding patterns

Congruent with our prediction 2, we found size‐specific differences in diet, but these were not always consistent across populations. Specifically, larger specimens were generally more likely to have consumed detritus, Cladocera, Diptera, Ostracoda, and Hemiptera. Our findings for the effect of SL on detritus consumption are in contrast with the results of Blanco et al., (2004). However, our other findings are in accordance with a study of Cabral et al., (1998), in which large eastern mosquitofish consumed a higher proportion of cladocerans and chironomids. Moreover, SL has shown to be an important factor for explaining differences in prey selection in many other fish species (Alcaraz & García‐Berthou, 2007; Mansfield & Mcardle, 1998; Jirka & Kraft, 2017). For instance, Alcaraz and García‐Berthou (2007) have reported larger Spanish toothcarp (*Aphanius iberus*) to prey on more benthic organisms than smaller specimens. Similar differences in feeding habits have been documented in a study of Mansfield and Mcardle (1998) on western mosquitofish. Specifically, smaller fish were found to have a greater preference for zooplankton, whereas larger specimens for larger invertebrates, albeit these differences were found to not be significant.

The size‐specific differences observed in our study could be driven by a variety of factors such as gape size and visual acuity, which have been suggested to play a role in intersize class differences in feeding behavior of the close relative *G. affinis (*Mansfield & Mcardle, 1998) as well as in a previous study on *G. holbrooki* (Singh & Gupta, 2010). Moreover, because eastern mosquitofish exhibit sexual size dimorphism with males being smaller than females (Bisazza, 1993), these size‐specific differences likely capture differences in feeding behavior between the sexes. This suggests that males are less likely to prey on detritus, Cladocera, Diptera, Ostracoda, and Hemiptera than females. This interpretation has been supported by previous studies (Blanco et al., 2004; Singh & Gupta, 2010). For instance, in an experimental study by Blanco et al., (2004), female *G. holbrooki* were observed to feed more intensively than males, and over a wider range of food items. Furthermore, females were found to prey more on zooplankton species, such as cladocerans, than males (Blanco et al., 2004).

With respect to the differences between populations, this is likely to be based on differences in abundance of Cladocera and Ostracoda between populations, as well as differences in habitat structure (for detritus, Coleoptera, and Araneae). Of particular note is this for the high occurrence of both Coleoptera and Araneae in the diet of population 9. This population was collected from a reed‐covered irrigation ditch surrounded by marsh and agricultural fields and we took note of the diversity of arthropods surrounding the habitat during sampling (F. Santi and R. Riesch, personal observation). This suggests that the high occurrence of both Coleoptera and Araneae in this particular population might indeed simply be the result of greater terrestrial arthropod abundance resulting in more opportunities for *G. holbrooki* to catch individuals that accidentally land or fall onto the water surface. However, we cannot exclude the possibility that these population differences might simply be a footprint of differential patterns of specialization between populations irrespective of patterns of local prey abundance.

Furthermore, we also uncovered a significant interaction of SL‐by‐population on the occurrence of detritus and Cladocera in the diet of mosquitofish, because occurrence increased with SL in some but decreased with SL in other populations. For Cladocera, this means that in three (Italian) populations, Cladocera were more likely to be eaten by larger fish (i.e., females), while males and small females were more likely to prey on Cladocera in the other two (Spanish) populations. For detritus, these contrasting patterns are more evenly spread across the sampled range, with larger fish (females) being more likely to have consumed detritus in five populations, while males and small females were more likely to have consumed detritus in three populations. These opposing patterns of size‐specific feeding ecology might be the result of differences in selective regimes, including differences in food availability, competition, or sexual selection. Regarding the pattern uncovered for Cladocera, we were able to show in previous work that fish from these Italian populations also differed strongly from those in the two Spanish populations in body condition and reproductive traits. Specifically, mosquitofish from these Italian populations exhibited higher levels of multiple paternity (Gao et al. 2019), and fish of both sexes were characterized by higher body fat content (Santi et al., 2020) compared to the two Spanish populations. However, while males from these Italian populations had greater gonadosomatic index compared to males from the two Spanish sites (also a proxy for the level of sperm competition), it was females from the two Spanish sites that exhibited a greater fecundity and invested more into reproduction (i.e., greater reproductive allocation; Santi et al., 2020). At present, we lack the data to properly address which specific selective factor(s) is/are driving these differences in (life histories and) size‐specific feeding ecology, but future research should investigate this further.

In addition, our investigation revealed an influence of fish SL on relative niche width, relative gut length and gut fullness. Specifically, smaller fish showed significantly narrower trophic niches, shorter relative gut lengths, and lower values of gut fullness. These results are consistent with the findings of previous studies on eastern mosquitofish and other fish species (Sánchez‐Hernández et al., 2012; Singh & Gupta, 2010). By contrast, in a study of Jirka and Kraft, (2017) the degree of individual specialization (i.e., niche width) of brook trout (*Salvelinus fontinalis*) was found to not differ as a function of fish length. As we argued above, these size differences likely largely reflect differences between the feeding behavior of males and females. Furthermore, there is evidence of females being foraging‐time maximizers with higher metabolic requirements and capacity to retain food longer compared to males who spend considerably more time in mating activities (Arrington et al., 2009; Pilastro et al., 2003). Concurrently, the finding of the level of gut fullness differing in relation to fish length may reflect the opportunistic behavior of this species. It is probable that to maximize the time spent in mating attempts and minimize the time spent feeding, smaller specimens (i.e., males) consume the prey items they come across to satisfy their daily energy requirements. A recent study of Singh and Gupta (2010) revealed an age‐dependent variation in gut fullness of *G. holbrooki*, with juveniles having higher values of GF than adults. Thus, future analysis of gut content might include variables such as age to further investigate this phenomenon.

Our analyses also revealed a significant effect of the interaction SL‐by‐population on relative gut length and gut fullness. Similar to our results for detritus, but in contrast to our results for Cladocera (see above), populations that did not conform to the overall trend of increasing RGL and GF with increasing SL were a mix of populations from Italy and Spain. Moreover, the subset of populations that had a negative association between SL and RGL was different from the subset of populations that had a negative association between SL and GF. None of the environmental or climatic variables we collected for each habitat help explain these differences, because there were no consistent differences in those variables that would set these habitats apart from the other habitats. In comparison to our results for Cladocera, there are also no obvious associations with life histories and patterns of multiple paternity (Gao et al., 2019; Santi et al., 2020). At present, we therefore do not have a convincing explanation for these results.

Dissecting these patterns raised an interesting *post hoc* question: Could there be an influence of female reproductive state on diet? Examining female‐specific GLMs with binomial family and logit link function that now parsed females into two categories based on the presence or absence of developing embryos (i.e., pregnant versus nonpregnant), we uncovered a significant effect of pregnancy on the presence/absence of Cladocera, with a higher likelihood of nonpregnant females having consumed Cladocera (*p*‐value < .01). No “pregnancy‐effect” was detected for any other prey category, nor was any effect of pregnancy found for relative niche width, gut fullness, and relative gut length (via GLMs with gamma family). This could be an indication that the abdominal distension resulting from pregnancy, which has been shown to negatively influence locomotor ability in a close relative, *G. affinis*, and other poeciliids (e.g., Ghalambor et al., 2004; Plaut, 2002), negatively affects foraging ability when chasing highly mobile prey such as Cladocera. Alternatively, this could be a result of changing habitat preferences for females that are pregnant. However, while behavioral change in response to pregnancy has been documented in a variety of taxa (e.g., reptiles: Bauwens & Thoen, 1981; Brodie, 1989), previous studies on the influence of reproductive state in mosquitofish found no evidence that pregnancy resulted in associated behavioral changes (*G. affinis*: Laidlaw et al., 2014), and to our knowledge, this is the first study that looked specifically at the effects of pregnancy on feeding habits. Future studies are therefore needed to further examine the potential effects of carrying young on mosquitofish feeding habits.

### 4.3 Influence of environmental, geographic, and climate variables on diet variety

Contrary to our prediction 3, we did not detect any effect of local environmental parameters, geographic and climatic variables on population differences in feeding patterns. Nonetheless, previous literature has documented that environmental parameters may strongly influence *G. holbrooki*’ feeding habits. For instance, contrary to our findings, a study of Cabral et al. (1998) revealed a positive correlation between number of prey ingested by eastern mosquitofish and water temperature and area covered by aquatic vegetation, while a reduction in the number of prey was observed with increasing pH and dissolved oxygen. It is plausible that we did not detect any effect of the local environmental conditions on population differences because the environmental variation between our sampling sites was simply not big enough to elicit such responses. For example, water pH varied between 6.3 and 9.4 across sites, but only at one sampling site was it greater than 7 (i.e., pH = 9.39 for site P3; Table A1). Additionally, some sampling sites, for example, P1 and P2, while being completely different in some environmental characteristics (with the first being a big lake, and the latter being a small stream nearby) were relatively close to each other, thus having very similar climates, further reducing variation in environmental parameters.

Other factors, which we did not quantify, could also help explain our results. Habitat characteristics such as percentage of vegetation cover and salinity could have contributed to the observed patterns given that previous work has documented these parameters influencing both feeding behavior and prey abundance in eastern mosquitofish (Cabral et al., 1998; Green et al., 2005). Additionally, population differences in feeding habits may also depend on other factors such as productivity (e.g., abundance of chlorophyll *a*), macroinvertebrate density, and seasonal shifts in prey use. Future studies should examine the contribution of these factors, potentially sampling specimens from the same populations over multiple seasons (e.g., winter and summer) and quantifying prey abundance in the water column using trap samples and at a larger geographic scale.

Finally, we did not find any effect of environmental, climatic, and geographic parameters on the proportion of specimens with empty guts. This could indicate that those individuals were simply less successful foragers and could point to limited resources in habitats with individuals with empty guts. However, we cannot exclude the possibility that these individuals had recently consumed something that was already fully digested at the time of sampling. Nonetheless, given that their invertebrate prey usually have hard exoskeletons, we find this last explanation less likely.

## 5 CONTRIBUTION TO MANAGEMENT

In addition to contributing considerably to the understanding of the feeding ecology of eastern mosquitofish, the results of this study may also be used by decision makers to design more effective management and control strategies for this highly invasive species. Thorough knowledge of mosquitofish feeding patterns is essential for an effective use of risk identification tools such as the Fish Invasiveness Scoring Kit (FISK), which is currently applied worldwide to mitigate the impact of invasions (e.g., Copp et al., 2009; Lawson et al., 2013). FISK classifies the risk of their introduction based on a variety of factors, including a fish's diet (e.g., whether the species is planktivorous or omnivorous).

Moreover, determining whether invasive species feed on endangered and/or threatened species is vital to devise effective conservation actions for those species as well as for the management of the invasive species feeding on them. Previous risk‐assessment investigations have implicated mosquitofish in the decline of native fish and anurans populations, some of which are important from a conservation perspective (e.g., green and golden bell frog, *Litoria aurea*: Remon et al., 2016; *Aphanius transgrediens*: Yoğurtçuoğlu & Ekmekçi, 2017). Nevertheless, we did not find any evidence of mosquitofish directly consuming any vertebrate species (e.g., fry, larvae or eggs) in our study even when sampling specimens across a wide range of habitats where mosquitofish co‐occur with native anurans (all sites) and several other fish species (e.g., Lago di Fimon in Italy or Rio Xuquer and Rio Vaca in Spain), including another killifish, *Aphanius fasciatus* (e.g., Marina di Grossetto, Italy). These findings may suggest that mosquitofish do not always pose a significant direct threat to amphibian and fish communities across their invasive range here in Europe. However, because our habitat‐specific sample sizes were relatively low (i.e., we investigated only an average of 20 fish per population), we cannot exclude the fact that *G. holbrooki* might be feeding on vertebrates also in our population but simply at very low incidence. Future studies are therefore needed to further examine competitive interaction between invasive mosquitofish and native species across this.

Finally, understanding the effects of environmental variables on mosquitofish feeding patterns is essential to understand the invasive potential of mosquitofish under current scenarios of climate change. Here, we found no evidence for an effect of temperature, despite water temperatures between habitats varying from 21.3 to 30.7°C and daily mean temperatures varying from 21.3 to 28.1°C (Table A1), and this was coupled with substantial dietary overlaps between populations. This suggests that mosquitofish feeding ecology may be less impacted by rising temperatures as a result from climate change compared to other freshwater fish species (Morgan et al., 2001; Snickars et al., 2015), probably partially as a result of their fairly wide dietary niche, ranging from algae and plants, via detritus, to invertebrates and vertebrates. In other words, global increasing temperatures as well as other climate change‐induced effects may actually exacerbate the competitive superiority of mosquitofish over native species (Rahel & Olden, 2008; Regmi et al., 2016).

## 6 CONCLUSION

Our results confirm that eastern mosquitofish are generalist predators, although they suggest an overall dietary preference for detritus and Cladocera. Size‐ and population‐specific differences in feeding patterns were documented and matched our predictions. In contrast, we did not detect any effect of environmental, geographic, and climatic parameters on population differences in diet. Our study provides a valuable contribution to knowledge on the feeding ecology of eastern mosquitofish in their invasive range, for the first time providing a direct comparison of dietary patterns in natural populations across a large geographic scale. In fact, our study reveals a large amount of flexibility in the diet of *G. holbrooki* (i.e., not a single diet item was shared by all populations), even though at least some taxonomic groups (like Cladocera and Diptera) were relatively common. This further helps explain the ubiquity of invasive mosquitofish across Italy and Spain. Being such flexible omnivores, they do not require the presence of one particular diet item in order to survive and establish a population, but can make use of whatever food might be locally abundant. Nonetheless, we still need a better understanding of what environmental and/or climatic features regulate the feeding patterns of this species. Thus, further enquiry into the ecological dimensions of different *Gambusia* habitats (e.g., the availability of local terrestrial and aquatic invertebrates, the structure of local food webs and the presence or absence as well as the exact nature of competitive interactions with other species) is critically needed to further deepen our understanding of why they are such successful invaders, and to identify and formulate correct management measures.

## CONFLICT OF INTEREST

None declared.

## AUTHOR CONTRIBUTIONS

**Sara Pirroni:** Data curation (lead); formal analysis (lead); investigation (equal); methodology (equal); visualization (lead); writing–original draft (lead); writing–review and editing (equal). **Laura de Pennafort Dezen:** Formal analysis (supporting); visualization (supporting); writing–review and editing (supporting). **Francesco Santi:** Conceptualization (equal); investigation (equal); writing–review and editing (supporting). **Ruediger Riesch:** Conceptualization (equal); investigation (equal); methodology (lead); project administration (lead); supervision (lead); validation (lead); writing–original draft (supporting); writing–review and editing (equal).

## ETHICAL STATEMENT

For the collection of these data, we have adhered to the Guidelines for the Use of Animals in Research. The study reported here is in agreement with the respective laws in Italy, Spain (Directive 2010/63/EU), and the UK [Animals (Scientific Procedures) Act 1986 Amendment Regulations (SI 2012/3039)], and all appropriate/necessary permits were at hand (please see Acknowledgments).

## Data Availability

All research data used for this project are available via Royal Holloway's Figshare data repository (https://doi.org/10.17637/rh.13675027).

## APPENDIX A

**TABLE A1:** List of the 9 sampling sites from which specimens of *Gambusia holbrooki* were collected including their location, number of fish caught (F: females; M: males), environmental parameters measured in the field (dissolved oxygen, pH, conductivity, water temperature), and site‐specific climate data (mean daily temperature, maximum and minimum daily temperature, and daily precipitation sum) downloaded from the ECA&D ver. 20.0e database at 0.1 degrees resolution (Cornes et al. 2018)

Population	Location	Sampling date	Latitude (N)	Longitude (E)	No. of samples (F\M)	Dissolved oxygen (mg/L)	pH	Conductivity (mS/cm)	Water temperature (°C)	Daily mean temperature (°C)	Daily minimum temperature (°C)	Daily maximum temperature (°C)	Daily precipitation sum (mm)
P1	Lago di Fimon N (IT)	27/07/2017	45.471	11.541	16 (10\6)	12.15	6.33	0.18	26.1	22.4	15.8	29.5	53.4
P2	Lago di Fimon S (IT)	27/07/2017	45.463	11.542	19 (10\9)	5.77	6.46	0.27	23.8	22.4	15.8	29.5	54.9
P3	Marina di Grosseto N (IT)	29/07/2017	42.731	10.963	19 (6\13)	7.26	9.39	4.02	24.5	26.1	22.6	37.1	35.1
P4	Marina di Grosseto S (IT)	30/07/2017	42.733	11.041	23 (10\13)	3.72	6.36	1.58	23.7	21.3	24.9	37.2	29.7
P5	El Palmar (ES)	07/08/2017	39.323	−0.320	18 (10\8)	2.53	6.44	1.63	28	25.9	21.2	31.9	54.9
P6	Río Xuquer (ES)	07/08/2017	39.177	−0.269	20 (10\10)	17.48	6.44	1.08	29.3	28.1	21.6	32.1	35.6
P7	Río Vaca (ES)	07/08/2017	39.061	−0.218	20 (10\10)	4.71	6.49	1.51	30.7	24.8	20.9	31.5	35.9
P8	Lebrija (ES)	10/08/2017	36.960	−6.064	16 (10\6)	7.64	6.40	5.63	21.3	25.5	13.7	32.1	93.2
P9	Doñana (ES)	10/08/2017	37.202	−6.262	12 (10\2)	6.28	6.38	2.65	26.7	25.8	15.9	34.6	85.5

**TABLE A2:** Description of how the different diet measurements and indices were calculated

Measurement	Description
Frequency of occurrence (FO)	$FO=\frac{N}{T}\times 100$ where *N* is the number of guts in which the prey items of one particular category are found and *T* is the total number of guts with food in the sample
Gut fullness (GF)	Fullness of the whole gut visually assessed and expressed as a percentage
Index of relative importance (IRI)	IRI=FO×(%N+%V) where *FO* is the frequency of occurrence of a prey category, *N* is the proportion of a certain food organism, and *V* is the food volume
Pianka overlap index	$O_{\mathrm{jk}}=\frac{\sum_{i}^{n}pijpik}{\sqrt{\sum_{i}^{n}pij^{2}\times \sum_{i}^{n}pik^{2}}}$ where *O_jk_* is Pianka's index of dietary overlap between the populations *j* and *k*, varying between 0 (no overlap) and 1 (complete overlap), *pij* represents the proportion of the *i* food resource in the diet of population *j*, *pik* is the proportion of the *i* food resource in the diet of population *k*, and *n* is the total number of prey items
Relative gut length (RGL)	$RGL\;=\;\frac{\mathrm{GL}}{\mathrm{SL}}$ where *GL* is the gut length (mm) and *SL* is fish standard length (mm)
Relative niche width (RNW)	$RNW\;=\;\frac{n_{i}}{N}$ where *RNW* is the relative niche width, *n* is the proportion of the food categories in the diet of the specimen *i,* and *N* is the total number of prey categories (including detritus). *RNW* ranges from 0 (specimen consumed items belonging to a single category) to 1 (specimen exploited all prey categories)
Schoener overlap index	$\alpha =1‐0.5\left(\sum_{i=1}^{n}\left|\;P_{\mathrm{xi}}‐P_{\mathrm{yi}}\right|\right)$ where *α* is the measure of the relative amount of dietary overlap, varying between 0 (no overlap) and 1 (complete overlap), *P_xi_* represents the proportion of food category *i* in the diet of the population *x*, *P_yi_* is the proportion of food category i in the diet of the population *y*, and *n* is the number of food categories
Shannon–Wiener index (*H*)	$H=‐\sum_{i=1}^{S}\left(pi\;\times \ln\;pi\right)$ where *H* is the Shannon–Wiener's index of prey diversity in the diet and *p* is the proportion of prey items of one particular food category *i* to the total number of prey categories found

**TABLE A3:** Principal component analysis (PCA) performed to reduce data dimensionality of environmental, climatic, and geographic data

Estimates	Principal components			
	PC1	PC2	PC3	PC4
Eigenvalue	3.097	2.410	2.293	1.412
Percentage of variance	30.969	24.102	22.931	14.123
Cumulative	30.969	55.070	78.002	92.124
Water temperature (°C)	−0.344	**0.753**	−0.393	0.253
Dissolved oxygen (mg/L)	0.003	0.020	−0.079	**0.914**
pH	0.171	0.104	**0.874**	0.202
Conductivity (mS/cm)	−0.500	−0.496	**0.661**	−0.112
Latitude (*N*)	**0.986**	0.065	−0.040	0.086
Longitude (E)	**0.964**	0.198	0.173	0.006
Daily mean temperature (°C)	**−0.762**	0.144	0.251	0.527
Daily maximum temperature (°C)	0.083	**0.862**	0.401	−0.195
Daily minimum temperature (°C)	−0.109	0.243	**0.803**	−0.360
Daily precipitation sum (mm)	−0.445	−0.849	−0.175	−0.080

Note

Shown are PCA axes and variable loadings for input variables. Bold values indicate the greatest and lowest loading for each principal component.

**TABLE A4:** Representation of the *p*‐values associated with multiple comparisons of relative gut length (RGL) between populations

	P1	P2	P3	P4	P5	P6	P7	P8	P9
P1	–								
P2	0.795	–							
P3	0.117	0.944	–						
P4	1.000	0.993	**0.017**	–					
P5	**<0.01**	**<0.01**	**<0.01**	**<0.01**	–				
P6	0.998	0.989	0.388	0.968	**<0.01**	–			
P7	1.000	0.855	0.126	0.999	**<0.01**	1.000	–		
P8	0.249	**<0.01**	**<0.01**	0.319	0.861	**0.026**	0.807	–	
P9	0.993	0.277	0.162	0.999	0.128	0.807	0.970	0.910	–

Note

Significant values are highlighted in bold.

**TABLE A5:** Representation of the *p*‐values associated with multiple comparisons of relative niche width (RNW) between populations

	P1	P2	P3	P4	P5	P6	P7	P8	P9
P1	–								
P2	**<0.001**	–							
P3	0.619	**0.028**	–						
P4	0.999	**<0.001**	0.903	–					
P5	0.999	**<0.001**	0.183	0.907	–				
P6	0.999	**<0.001**	0.161	0.901	1.000	–			
P7	1.000	**<0.001**	0.785	1.000	0.982	0.982	–		
P8	0.853	**0.013**	1.000	0.989	0.422	0.403	0.952	–	
P9	0.996	**<0.001**	0.327	0.948	1.000	1.000	0.990	0.550	–

Note

Significant values are highlighted in bold.

**TABLE A6:** Representation of the *p*‐values associated with multiple comparisons of gut fullness (GF) between populations

	P1	P2	P3	P4	P5	P6	P7	P8	P9
P1	–								
P2	**<0.01**	–							
P3	0.972	**<0.01**	–						
P4	1.000	**<0.01**	0.929	–					
P5	0.823	**<0.01**	0.122	0.789	–				
P6	0.928	**<0.01**	0.205	0.912	1.000	–			
P7	0.763	**<0.01**	0.081	0.710	1.000	1.000	–		
P8	0.987	**<0.01**	1.000	0.967	0.192	0.308	0.141	–	
P9	0.977	**<0.01**	0.470	0.978	1.000	1.000	1.000	0.547	–

Note

Significant values are highlighted in bold.

**TABLE A7:** Parameter estimates of the linear regression models investigating the influence of environmental, climatic, and geographic parameters on Diversity index, frequency of occurrence (FO), and index of relative importance (IRI)

	Estimate	*SE*	*z*	*p*
Diversity index model				
Intercept	0.36	0.09	4.01	.02
PC1	−0.00	0.00	−0.04	.97
PC2	0.00	0.00	−0.06	.96
PC3	0.00	0.00	−0.19	.86
PC4	0.00	0.00	0.61	.58
FO model				
Intercept	119.48	17.84	6.70	.00
PC1	−0.35	0.23	−1.54	.20
PC2	0.08	0.21	0.38	.72
PC3	−0.02	0.22	−0.07	.95
PC4	0.11	0.21	0.51	.64
IRI model				
Intercept	34,853.79	12,632.77	2.76	.07
PC1	−118.92	187.08	−0.64	.57
PC2	168.46	141.48	1.19	.32
PC3	−94.95	151.73	−0.63	.57
PC4	−19.86	142.11	−0.14	.90

Note

Environmental, climatic, and geographic parameters were incorporated using principal component analysis (PCA).

**TABLE A8:** Parameter estimates of the linear regression models investigating the influence of environmental, climatic, and geographic parameters on the total amount of prey ingested by eastern mosquitofish

	Estimate	*SE*	*z*	*p*
Intercept	189.89	115.39	1.646	.175
PC1	−95.79	122.39	−0.783	.478
PC2	173.18	122.39	1.415	.230
PC3	−107.88	122.39	−0.881	.428
PC4	−22.72	122.39	−0.186	.862

Note

Environmental, climatic, and geographic parameters were incorporated using principal component analysis (PCA).

**TABLE A9:** Parameter estimates of the linear regression models investigating the influence of environmental, climatic, and geographic parameters on the proportion of specimens with empty guts

	Estimate	*SE*	*z*	*p*
Intercept	50.378	11.188	4.503	.010
PC1	−14.975	11.866	−1.262	.275
PC2	8.432	11.866	0.711	.517
PC3	−16.338	11.866	−1.377	.241
PC4	−25.464	11.866	−2.146	.098

Note

Environmental, climatic, and geographic parameters were incorporated using principal component analysis (PCA).
